# Deep learning diagnostic and risk-stratification pattern detection for COVID-19 in digital lung auscultations: clinical protocol for a case–control and prospective cohort study

**DOI:** 10.1186/s12890-021-01467-w

**Published:** 2021-03-24

**Authors:** Alban Glangetas, Mary-Anne Hartley, Aymeric Cantais, Delphine S. Courvoisier, David Rivollet, Deeksha M. Shama, Alexandre Perez, Hervé Spechbach, Véronique Trombert, Stéphane Bourquin, Martin Jaggi, Constance Barazzone-Argiroffo, Alain Gervaix, Johan N. Siebert

**Affiliations:** 1 Division of Paediatric Emergency Medicine, Department of Women, Child and Adolescent, Geneva University Hospitals, 47 Avenue de la Roseraie, 1205 Geneva, Switzerland; 2 Intelligent Global Health, Machine Learning and Optimization (MLO) Laboratory, Swiss Federal Institute of Technology (EPFL), Lausanne, Switzerland; 3 Quality of Care Unit, Geneva University Hospitals, Geneva, Switzerland; 4 Essential Tech Centre, Swiss Federal Institute of Technology (EPFL), Lausanne, Switzerland; 5 Geneva University Hospitals, Geneva, Switzerland; 6 Division of Primary Care Medicine, Department of Community Medicine, Geneva University Hospitals, Geneva, Switzerland; 7 Department of Internal Medicine and Rehabilitation, Geneva University Hospitals, Geneva, Switzerland; 8 Department of Micro-Engineering, Geneva School of Engineering, Architecture and Landscape (HEPIA), Geneva, Switzerland; 9 Paediatric Pulmonology Unit, Department of Women, Child and Adolescent, University Hospitals of Geneva, Geneva, Switzerland

**Keywords:** COVID-19, Severe acute respiratory syndrome coronavirus 2 (SARS-CoV-2), Deep learning, Artificial intelligence, Respiratory sounds, Auscultation, Pneumonia

## Abstract

**Background:**

Lung auscultation is fundamental to the clinical diagnosis of respiratory disease. However, auscultation is a subjective practice and interpretations vary widely between users. The digitization of auscultation acquisition and interpretation is a particularly promising strategy for diagnosing and monitoring infectious diseases such as Coronavirus-19 disease (COVID-19) where automated analyses could help decentralise care and better inform decision-making in telemedicine. This protocol describes the standardised collection of lung auscultations in COVID-19 triage sites and a deep learning approach to diagnostic and prognostic modelling for future incorporation into an intelligent autonomous stethoscope benchmarked against human expert interpretation.

**Methods:**

A total of 1000 consecutive, patients aged ≥ 16 years and meeting COVID-19 testing criteria will be recruited at screening sites and amongst inpatients of the internal medicine department at the Geneva University Hospitals, starting from October 2020. COVID-19 is diagnosed by RT-PCR on a nasopharyngeal swab and COVID-positive patients are followed up until outcome (i.e., discharge, hospitalisation, intubation and/or death). At inclusion, demographic and clinical data are collected, such as age, sex, medical history, and signs and symptoms of the current episode. Additionally, lung auscultation will be recorded with a digital stethoscope at 6 thoracic sites in each patient. A deep learning algorithm (*DeepBreath*) using a Convolutional Neural Network (CNN) and Support Vector Machine classifier will be trained on these audio recordings to derive an automated prediction of diagnostic (COVID positive vs negative) and risk stratification categories (mild to severe). The performance of this model will be compared to a human prediction baseline on a random subset of lung sounds, where blinded physicians are asked to classify the audios into the same categories.

**Discussion:**

This approach has broad potential to standardise the evaluation of lung auscultation in COVID-19 at various levels of healthcare, especially in the context of decentralised triage and monitoring.

*Trial registration*: PB_2016-00500, SwissEthics. Registered on 6 April 2020.

**Supplementary Information:**

The online version contains supplementary material available at 10.1186/s12890-021-01467-w.

## Background

Since the invention of the stethoscope by René-Théophile-Hyacinthe Laennec in the nineteenth century, the interpretation of internal body sounds has made the stethoscope a ubiquitous part of a doctor’s uniform. As stethoscopes are easy to handle, inexpensive and non-invasive, they can provide valuable clinical information at any level of care, and are a fundamental step in the preliminary clinical diagnosis and early assessment of pulmonary disease to shorten delays in diagnosis and emergency management [1]. However, the utility of this tool is ultimately dependent on the user’s perceptual capacity to discriminate and interpret pathological patterns in sound across the respiratory cycle. Follow-up comparative assessments are then based on the user’s ability to remember these patterns from a previous time point, or mentally reconstruct them from descriptions written in clinical notes. As this interpretation is a highly subjective skill, inter-listener variability limits interoperability, where accuracy ranges widely with experience and differs across specialities [2, 3]. Discrepancies also arise due to a lack of standardisation in nomenclature [3] rendering the results equivocal and/or incomprehensible to other caregivers. Other sources of heterogeneity may originate from differences in the intrinsic properties of the stethoscope and extrinsic patient-related factors such as obesity, ambient noise and patient compliance (e.g., crying child).

To better standardise the detection of abnormal lung sounds, there has been a recent interest in digitizing respiratory sound acquisition using electronic stethoscopes [4–7] and then analysing it using artificial intelligence (AI) methods of deep learning [8–13]. For the COVID-19, caused by severe acute respiratory syndrome coronavirus 2 (SARS-CoV-2), AI methods have revealed clear patterns in its radiological presentation [14–19], and some preliminary evidence on the predictive capacity of respiratory sound is emerging. For instance, the application of simple models such as logistic regression and support vector machine (SVM) were able to predict the diagnosis of COVID-19 from breath and cough sounds crudely collected on a mobile application with an area under the curve (AUC) of around 70% [20]. Another group achieved above 95% sensitivity and specificity on discriminating COVID-19 coughs from other pathologies as well as healthy patients [21]. However, no evidence exists for the potential of digital lung sounds for early detection and, more importantly, risk stratification in COVID-19. Indeed, while around 80% of infections are either asymptomatic or self-resolving after a few weeks of mild disease, the remaining 20% can rapidly progress to acute respiratory distress syndrome with a poor prognosis and high mortality [22, 23]. Hence, early risk stratification is crucial for prompt referral and early intervention, as well as the appropriate allocation of limited hospital resources.

Owing to the initial non-specific symptomatic presentation of COVID-19, diagnosis and risk stratification are based on more objective paraclinical exams, such as reverse transcription polymerase chain reaction (RT-PCR) to detect the genetic signatures of SARS-CoV-2 in nasal-pharyngeal swabs for diagnosis, and high-resolution chest computerized tomography (CT) to estimate prognosis [24, 25]. However, even RT-PCR may yield false-negative results [26] and in times of high transmission, testing backlogs can render turn-around times clinically irrelevant [27]. On the other hand, risk stratification by CT is inconvenient for a variety of reasons. Firstly, these machines are usually housed in centralised high-level healthcare infrastructures, inappropriate for triage, and necessitate the transfer/handling of potentially infectious patients. Secondly, they expose patients to ionizing radiation and, thirdly, the cost and skill required to acquire and use these machines have made CT scanners rarely available in many parts of the world.

Thus, we aim to develop a set of early diagnostic and risk-stratification algorithms for COVID-19 from lung auscultations. To this end, we will collect standardised digital lung recordings from patients triaged for COVID-19 testing at screening sites or already hospitalised for diagnosed COVID-19. We hypothesize that early diagnostic and prognostic acoustic signatures of COVID-19 can be detected by deep learning independently of the caregiver’s auscultation skills, thus better standardising decision making and resource allocation.

## Methods

### Study design

This is a single-centre population-based study divided into two aims; a case–control study for diagnostic classification, and a prospective cohort study for risk stratification. For diagnosis, cases will be those testing positive for COVID-19 by RT-PCR for SARS-CoV-2 virus on a nasopharyngeal swab (sensitivity: 89.0%, specificity: 99.70%) [28] at any time during the 14 days following enrolment. Thus, if a patient is initially classified as COVID-negative and becomes positive within 14 days of enrolment the initial test result is considered false-negative and the patient is then re-classified as COVID-positive. Controls will be those presenting at triage who consistently receive negative COVID-test results during the 14 days following their enrolment. In addition to triage sites, COVID-positive hospitalised patients (not in intensive care) will also be enrolled. RT-PCR tests will be performed and repeated according to public health guidelines, described in Table 1. For risk stratification, COVID-positive patients will be separated into ordinal severity classes shown in Table 2.

**Table 1:** Study schedule

Time point	t_0_	Day 1	Each 48 h	Day of outcome^a^
Study procedures				
Recruitment	✓			
Eligibility screening	✓			
Informed consent form	✓			
Assessments				
Case report form (Demographic + clinical data)	✓			
RT-PCR SARS-CoV-2 test (sensitivity)	✓	(✓)^b^	(✓)^b^	
Digital auscultation collection				
COVID-positive recruited in triage sites	✓	(✓)^c^	(✓)^c^	(✓)^c,d^
COVID-positive recruited as inpatients	✓	✓	✓	(✓)^d^
COVID-negative patients	✓			

✓: performed, (✓): performed with stipulated conditions

^a^If recruited in triage sites: (1) No hospitalisation within 7 days of enrolment, (2) hospitalisation within 7 days of enrolment with discharge, (3) hospitalisation within 7 days of enrolment with ICU admission/death

If recruited as inpatients: (1) Discharge with without worsened condition attributable to COVID-19, (2) Worsened condition attributable to COVID-19 with ICU admission/death

^b^If initially negative and remains symptomatic with renewed testing criteria

^c^If later hospitalised

^d^Not collected once admitted to intensive care

**Table 2:** Outcome groups

Diagnostic outcome			Risk stratification outcomes	
			Triage patients COVID+	Hospitalised patients COVID+
COVID+	Any positive RT-PCR for SARS-CoV-2 on a nasopharyngeal swab during the 14 days following enrolment	Mild	Outpatients discharged home with mild disease without hospitalisation within 7 days of enrolment	Patients without worsening condition attributable to COVID-19 and discharged home
		Moderate	Patients hospitalised within 7 days of enrolment and NOT receiving intensive care at any point during their hospitalisation	Patients with worsening condition attributable to COVID-19 requiring ICU referral/or dying during follow-up
		Severe	Patients hospitalised within 7 days of enrolment and subsequently requiring ICU referral/or dying	
COVID-	Consistently negative RT-PCR for SARS-CoV-2 on a nasopharyngeal swab during the 14 days following enrolment			

### Population

Inclusion criteria are consecutive consenting adult patients (i.e. aged ≥ 16 years) meeting COVID-19 testing criteria. At the time of writing, testing criteria are having been in close contact (less than 1.5 m for a total of 15 min or more) with documented SARS-CoV-2 infection, and/or having any of the following symptoms: cough, dyspnoea, fever, sudden loss of taste/smell, flu-like symptoms (i.e. sore throat, runny or stuffy nose, muscle or body aches, headaches, fatigue, etc.) [29]. Exclusion criteria are: (1) oxygen supplementation greater than 10L/min delivered by any device (due to major modifications of the auscultatory sounds), (2) mechanically ventilated patients (due to major modifications of the auscultatory sounds), (3) severely ill patients hospitalised in intensive care units, (4) patients who cannot be mobilised for posterior auscultation, (5) patients known or suspected of immunodeficiency, and or under immunotherapy. Due to the non-specific symptoms of COVID, the negative patients comprise a vast range of differential diagnoses which are not recorded as we rather aim represent all patients attending a screening facility irrespective of the differential diagnosis.

### Data collection

For each patient, demographic and clinical data will be collected including, age, sex, medical history, pre-existing diseases known to predispose poor outcomes in COVID-19 (according to the US Centers for Disease Control and Prevention [30]), and signs and symptoms of the current episode. Lung auscultations will be recorded with a Littmann 3200 electronic stethoscope (3 M Health Care, St. Paul, USA) using the Littmann StethAssist proprietary software v.1.3. Digital lung auscultations will be performed at 6 thoracic sites for at least 30 s each. The sites are four posterior (left and right apical and basal zones) and 2 axillary sites (right, left). Anterior sites will not be auscultated to both reduce the interference of heart sounds and prevent the airborne transmission of SARS-CoV-2 to investigators. The audio files and patient clinical data will be encoded as anonymised files by the local investigators and uploaded to the RedCap server hosted by the hospital (REDCap, Vanderbilt University, Nashville, TN, USA; https://www.project-redcap.org/resources/citations/). Patients positive for COVID-19 will then be followed up until outcome (i.e., discharge, hospitalisation, intubation and/or death, which serve as labels for prognostic risk stratification). Hospitalised patients will have repeat lung auscultations each 48 h until discharge, ICU referral or death. The study schedule is detailed in Table 1.

### Sample size calculation

*Deep learning algorithm derivation*: will be used to decompose the audio signals into meaningful parameters. Each patient will provide 6 recordings of 30 s each. The sample size considerations are estimated for the train and test sets in such a way that performance (sensitivity, specificity, area under the receiver operating characteristic [ROC] curve and accuracy) could be derived with minimal variance on a stable training curve.

**Fig. 1:**
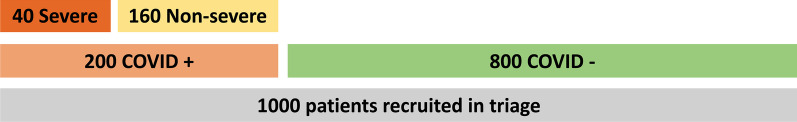
Sample size required to secure at least 40 patients in each outcome group

Assuming a similar discriminative power compared to a previous work (personal communications) distinguishing healthy and pathological lung sounds in pneumonia from 80 patients in balanced classes (40 pathological and 40 control) with 8 auscultation sites of 30 s each, we estimate to achieve convergence at above 80% AUC-ROC with 10% variability using the same number of patients in each class (i.e. for diagnosis: 40 COVID positive and 40 COVID negative, and for risk stratification: 40 severe and 40 non-severe). Starting with the requirement for risk stratification of 40 COVID-positive patients with severe disease: 20% of COVID-positive patients are expected to be classified as “severe” (with hospitalisation required) [31]. We would thus require at least 200 COVID-positive patients to be recruited. As the expected positivity rate for patients at this recruitment sites averages around 20%, we would require at least 1000 enrolments to secure 200 COVID-positive patients (Fig. 1).

Currently there are around 4900 tests per week in the targeted outpatient group in Geneva University Hospitals. Thus, with a test positivity rate of 11.4% as of November 11^th^ 2020 [32], we would have 4341 COVID-negative and 559 COVID-positive patients per week, meaning that recruitment should take one week. Assuming 50% recruitment consent and accounting the lability of these epidemiological values, we anticipate a recruitment period of 1–2 months.

### Human predictive baseline

In order to generate a human baseline for sound-based diagnostic and risk stratification interpretation, randomly selected lung sounds will be blinded for outcomes and randomly evaluated by several clinicians (residents, fellows, professors and pulmonologists) who will be required to classify them as either COVID-positive or COVID-negative (with and without the availability of their clinical and demographic information). Once this is completed, the clinicians will be given only the set of COVID-positive samples and asked to stratify their risk (with and without the availability of their clinical symptomology and demographic information collected on day 0 before any diagnostic tests are undertaken). Lung sounds will also be annotated by expert consensus for audible pathology types (wheezing, crackles, rhonchi, etc.).

A kappa statistic for interrater concordance will be computed for each evaluation and a ROCAUC reported according to the outcome. The distribution of discriminative sound patterns identified by the human clinical experts will be explored by unsupervised machine learning using clustering on k-means (grouping sounds into k number of clusters, which will be further optimized using the Elbow method). Thus, objective sound patterns will be clustered by k-means in a vector space [33] and the distribution of human labels within these clusters will be reported to visualise the alignment between objective patterns and human interpretation.

### The development of DeepBreath

DeepBreath is a deep learning algorithm to diagnose and stratify risk of COVID-19 from lung sounds. In preparation for these analyses, digital auscultations are cleaned to crop non-biological frequencies and amplitudes generated by ambient noise. The frequency range of normal lung sounds extends from below 100 Hz to 1000 Hz, with a sharp drop at approximately 100 to 200 Hz [1], whereas tracheal sound extends between 100 and 5000 Hz. In the lower band range (under 100 Hz), heart and thoracic muscle sounds overlap [34]. Abnormal lung sounds (wheezing, rhonchi etc.) have characteristic frequencies and duration, differentiating them from each other [1]. In this study, all signals will be sampled at 16 kHz with a resolution of 8-bit, and the built-in filter will range from 20 to 2000 Hz. Heart and thoracic muscle sounds, as well as other background low-frequency noises will be filtered out through a high-pass filter (cut-off frequency 150 Hz).

The sounds will then be divided into overlapping time windows of between 1 and 10 s and transformed to Mel Frequency Cepstral Coefficients (MFCCs). Several data augmentation techniques will be explored, such as amplitude scaling, pitch shift, and random time shift. The effect of each pre-processing method will be tested and the best performing approach according to sensitivity and specificity will be reported. This dataset will then be fed into a convolutional neural network with max pooling and dropout before binary classification by a support vector machine (SVM) into positive vs negative COVID-19 test results (diagnostic model) or hospitalisation/death vs outpatient/self-resolving (risk stratification model). Longitudinal auscultations on individual patients who were hospitalised will also be used to assess the severity and progression of the disease, normalized at the time of symptom onset.

### Statistical analysis plan

Firstly, the collected data will be described. Continuous variables will be reported as means with their 95% confidence intervals (CI). Categorical variables will be reported as proportions and percentages. Features will be compared between outcome groups for diagnosis (COVID-positive versus COVID-negative) and prognosis (within COVID positive: outpatient, hospitalisation, worsening patients requiring ICU referral/death) using logistic regression, with risk ratios and CI95%. Pearson’s and Spearman’s correlation coefficients will be used to assess the relationship between continuous variables normally and non-normally distributed, respectively.

Missing data will be padded with zero in the CNN and reported in the descriptive statistics. Missingness will also be assessed according to other features. Features with more than 50% missing values or with significant bias in missingness will be removed. For the primary outcome, the ability of the DeepBreath algorithm to distinguish between COVID-19 positive and negative patients as well as between severity outcome groups will be quantified using the area under the receiver operating characteristic curve (AUC), sensitivity, specificity, positive predictive value, negative predictive value, and likelihood ratios, presented with their 95% CI. The accuracy of the AI algorithm will be compared with human expert discrimination by sound. The optimism of the AI algorithm will be estimated by the difference in performance between the training and validation sets.

All statistical tests will be two-sided with a type-I error risk of 5%. Data analysis will be carried out using GraphPad Prism, version 9 (GraphPad Software, San Diego, CA, USA) for graph figures, and R version 4.0 (R Foundation, Vienna, Austria) for descriptive statistics and statistical tests.

## Discussion

The recent advances in deep learning are promising to support physicians in standardizing the detection and interpretation of complex patterns in pulmonary diseases. Artificial intelligence has proven to outperform physicians in discriminating respiratory pathologies via respiratory functional explorations [35], symptoms [36, 37], and/or radiological examinations [38]. The development of AI algorithms for the analysis of respiratory acoustic signals has been proposed previously [9, 10]. It remains to be determined whether an AI algorithm can be used as an initial and accurate screening tool for patients suspected of COVID-19. Such an algorithm has the potential to support early diagnosis, to guide the allocation of resources and identify those in need of early hospitalisation [39].

This study aims to collect a standardised dataset of digital lung auscultations and derive a deep leaning model able to detect the acoustic signatures of the presence and severity of COVID-19. We hypothesize that automated interpretation of lung auscultation could better democratize the accuracy of this critical clinical exam beyond the individual capabilities of the health workers that a patient may be fortunate (or unfortunate) to have. We plan to incorporate this algorithm into an autonomous digital stethoscope (currently under development), that could help decentralise high quality respiratory examination and monitoring, and perhaps even empower patients to assess themselves, which would reduce nosocomial infections occurring during the proximity of a traditional clinical exam. Making high quality lung auscultation accessible to unskilled/decentralized actors could broaden COVID-19 mass screening and identify patients at earlier stages of disease, which would prevent transmission as well as allow earlier pharmacological interventions and nuance the pre-test probability to better select candidates for further PCR testing (i.e. resource conservation). Additionally, such a tool could empower patients confined at home to self-monitor their symptoms to inform telemedicine and personalize care.

This study has several limitations. First, ICU-inpatients with severe COVID-19 presentations requiring ventilatory supports will not be considered. This will limit longitudinal auscultations on critically-ill patients and prevent risk stratification assessment of those who will progress very unfavourably (or favourably) from this stage onwards. Second, since this study is a single centre study conducted in a high-income country with easy access to health care, caution will be taken when assessing the generalisability of these results to different populations, especially in resource-limited countries and remote areas. Finally, our study population will be recruited primarily from an emergency department triage centre and COVID-19-dedicated hospitalization units, which may suggest more acute symptoms and pathological lung sounds than those encountered in ambulatory care services.

## Supplementary Information

**Additional file 1.** STROBE Statement checklist.

## Data Availability

An anonymous copy of the final (anonymised) datasets used and/or analysed during the current study will be available from the corresponding author on reasonable request. All pertinent data generated or analysed during this study will included in the published article [and its supplementary information files].

## Abbreviations

AI: Artificial Intelligence
AUC: Area under the curve
CNN: Convolutional Neural Network
COVID-19: Coronavirus-19 disease
CT: Computerized Tomography
MFCC: Mel-Frequency Cepstral Coefficients
ROC: Receiver Operating Characteristic
RT-PCR: Reverse Transcription Polymerase Chain Reaction
SARS-CoV-2: Severe Acute Respiratory Syndrome Coronavirus 2
SVM: Support Vector Machine

## Declarations

### Study status

The protocol version is 1.0 (20 November 2020).

## References

[1] Bohadana A, Izbicki G, Kraman SS (2014). Fundamentals of lung auscultation. N Engl J Med.

[2] Sarkar M, Madabhavi I, Niranjan N, Dogra M (2015). Auscultation of the respiratory system. Ann Thorac Med.

[3] Hafke-Dys H, Breborowicz A, Kleka P, Kocinski J, Biniakowski A (2019). The accuracy of lung auscultation in the practice of physicians and medical students. PLoS ONE.

[4] Andres E, Gass R, Charloux A, Brandt C, Hentzler A (2018). Respiratory sound analysis in the era of evidence-based medicine and the world of medicine 2.0. J Med Life.

[5] Gurung A, Scrafford CG, Tielsch JM, Levine OS, Checkley W (2011). Computerized lung sound analysis as diagnostic aid for the detection of abnormal lung sounds: a systematic review and meta-analysis. Respir Med.

[6] Pinho C, Oliveira A, Jácome C, Rodrigues JM, Marques A. Integrated approach for automatic crackle detection based on fractal dimension and box filtering. In: Data analytics in medicine: concepts, methodologies, tools, and applications. edn. Edited by Global I; 2020: 815–832.

[7] Pancaldi F, Sebastiani M, Cassone G, Luppi F, Cerri S, Della Casa G, Manfredi A (2018). Analysis of pulmonary sounds for the diagnosis of interstitial lung diseases secondary to rheumatoid arthritis. Comput Biol Med.

[8] Abbas A, Fahim A (2010). An automated computerized auscultation and diagnostic system for pulmonary diseases. J Med Syst.

[9] Grzywalski T, Piecuch M, Szajek M, Breborowicz A, Hafke-Dys H, Kocinski J, Pastusiak A, Belluzzo R (2019). Practical implementation of artificial intelligence algorithms in pulmonary auscultation examination. Eur J Pediatr.

[10] Palaniappan R, Sundaraj K, Sundaraj S (2014). Artificial intelligence techniques used in respiratory sound analysis–a systematic review. Biomed Tech (Berl).

[11] Bhatt SP, Washko GR, Hoffman EA, Newell JD, Bodduluri S, Diaz AA, Galban CJ, Silverman EK, San Jose Estepar R, Lynch DA (2019). Imaging advances in chronic obstructive pulmonary disease Insights from the Genetic Epidemiology of Chronic Obstructive Pulmonary Disease (COPDGene) Study. Am J Respir Crit Care Med.

[12] Das N, Topalovic M, Janssens W (2018). Artificial intelligence in diagnosis of obstructive lung disease: current status and future potential. Curr Opin Pulm Med.

[13] Mlodzinski E, Stone DJ, Celi LA (2020). Machine learning for pulmonary and critical care medicine: a narrative review. Pulm Ther.

[14] Zhang K, Liu X, Shen J, Li Z, Sang Y, Wu X, Zha Y, Liang W, Wang C, Wang K, et al. Clinically applicable AI system for accurate diagnosis, quantitative measurements, and prognosis of COVID-19 pneumonia using computed tomography. *Cell* 2020, 181(6):1423–1433 e1411. 10.1016/j.cell.2020.04.045 PMC7196900 32416069

[15] Liu F, Zhang Q, Huang C, Shi C, Wang L, Shi N, Fang C, Shan F, Mei X, Shi J (2020). CT quantification of pneumonia lesions in early days predicts progression to severe illness in a cohort of COVID-19 patients. Theranostics.

[16] Singh D, Kumar V (2020). Vaishali, Kaur M: Classification of COVID-19 patients from chest CT images using multi-objective differential evolution-based convolutional neural networks. Eur J Clin Microbiol Infect Dis.

[17] Apostolopoulos ID, Mpesiana TA (2020). Covid-19: automatic detection from X-ray images utilizing transfer learning with convolutional neural networks. Phys Eng Sci Med.

[18] Ko H, Chung H, Kang WS, Kim KW, Shin Y, Kang SJ, Lee JH, Kim YJ, Kim NY, Jung H (2020). COVID-19 pneumonia diagnosis using a simple 2D deep learning framework with a single chest CT image: model development and validation. J Med Internet Res.

[19] Ozturk T, Talo M, Yildirim EA, Baloglu UB, Yildirim O, Rajendra Acharya U (2020). Automated detection of COVID-19 cases using deep neural networks with X-ray images. Comput Biol Med.

[20] Brown C, Chauhan J, Grammenos A, Han J, Hasthanasombat A, Spathis D, Xia T, Cicuta P, Mascolo C. Exploring automatic diagnosis of COVID-19 from crowdsourced respiratory sound data. arXivorg 2020.

[21] Imran A, Posokhova I, Qureshi HN, Masood U, Riaz MS, Ali K, John CN, Hussain MI, Nabeel M (2020). AI4COVID-19: AI enabled preliminary diagnosis for COVID-19 from cough samples via an app. Inform Med Unlocked.

[22] National Center for Immunization and Respiratory Diseases (U.S.). Division of Viral Diseases. Centers for Disease Control and Prevention: Interim Clinical Guidance for Management of Patients with Confirmed Coronavirus Disease (COVID-19). Updated June 30, 2020. https://stacks.cdc.gov/view/cdc/89980. Accessed 15 Nov 2020.

[23] Li X, Ma X (2020). Acute respiratory failure in COVID-19: is it "typical" ARDS?. Crit Care.

[24] Ai T, Yang Z, Hou H, Zhan C, Chen C, Lv W, Tao Q, Sun Z, Xia L. Correlation of chest CT and RT-PCR testing in coronavirus disease 2019 (COVID-19) in China: a report of 1014 cases. Radiology 2020:200642. 10.1148/radiol.2020200642 PMC7233399 32101510

[25] Salaffi F, Carotti M, Tardella M, Borgheresi A, Agostini A, Minorati D, Marotto D, Di Carlo M, Galli M, Giovagnoni A (2020). The role of a chest computed tomography severity score in coronavirus disease 2019 pneumonia. Medicine (Baltimore).

[26] Kucirka LM, Lauer SA, Laeyendecker O, Boon D, Lessler J (2020). Variation in false-negative rate of reverse transcriptase polymerase chain reaction-based SARS-CoV-2 tests by time since exposure. Ann Intern Med.

[27] Francone M, Iafrate F, Masci GM, Coco S, Cilia F, Manganaro L, Panebianco V, Andreoli C, Colaiacomo MC, Zingaropoli MA (2020). Chest CT score in COVID-19 patients: correlation with disease severity and short-term prognosis. Eur Radiol.

[28] Kaiser L, Schibler M, Berger A, Eckerle I: Validation report: SARS-CoV-2 antigen rapid diagnostic test. https://www.hug.ch/sites/interhug/files/structures/laboratoire_de_virologie/documents/Centre_maladies_virales_infectieuses/ofsp_rdt_report_gcevd_27.10.2020.pdf*.* Accessed 9 Feb 2021.

[29] Centers for Disease Control and Prevention: Symptoms of coronavirus. https://www.cdc.gov/coronavirus/2019-ncov/symptoms-testing/symptoms.html. Accessed 15 Nov 2020.

[30] Centers for Disease Control and Prevention: People with certain medical conditions. https://www.cdc.gov/coronavirus/2019-ncov/need-extra-precautions/people-with-medical-conditions.html. Accessed 15 Nov 2020.

[31] Karagiannidis C, Mostert C, Hentschker C, Voshaar T, Malzahn J, Schillinger G, Klauber J, Janssens U, Marx G, Weber-Carstens S (2020). Case characteristics, resource use, and outcomes of 10 021 patients with COVID-19 admitted to 920 German hospitals: an observational study. Lancet Respir Med.

[32] Swiss Federal Office of Public Health: Situation report on the epidemiological situation in Switzerland and the Principality of Liechtenstein. https://www.bag.admin.ch/bag/fr/home/krankheiten/ausbrueche-epidemien-pandemien/aktuelle-ausbrueche-epidemien/novel-cov/situation-schweiz-und-international.html#-1315239417. Accessed 13 Nov 2020.

[33] Franceschi J-Y, Dieuleveut A, Jaggi M. Unsupervised scalable representation learning for multivariate time series. arXiv 2020: eprint 1901.10738.

[34] Sanchez I, Vizcaya C (2003). Tracheal and lung sounds repeatability in normal adults. Respir Med.

[35] Topalovic M, Das N, Burgel PR, Daenen M, Derom E, Haenebalcke C, Janssen R, Kerstjens HAM, Liistro G, Louis R (2019). Artificial intelligence outperforms pulmonologists in the interpretation of pulmonary function tests. Eur Respir J.

[36] Bardou D, Zhang K, Ahmad SM (2018). Lung sounds classification using convolutional neural networks. Artif Intell Med.

[37] Chamberlain D, Kodgule R, Ganelin D, Miglani V, Fletcher RR (2016). Application of semi-supervised deep learning to lung sound analysis. Conf Proc IEEE Eng Med Biol Soc.

[38] Walsh SLF, Calandriello L, Silva M, Sverzellati N (2018). Deep learning for classifying fibrotic lung disease on high-resolution computed tomography: a case-cohort study. Lancet Respir Med.

[39] Ting DSW, Carin L, Dzau V, Wong TY (2020). Digital technology and COVID-19. Nat Med.

[40] World Medical A (2013). World Medical Association Declaration of Helsinki: ethical principles for medical research involving human subjects. JAMA.

[41] International Conference on Harmonisation: ICH Harmonised Tripartite Guideline. Statistical principles for clinical trials. International Conference on Harmonisation E9 Expert Working Group. Stat Med 1999, 18(15):1905–1942. 10532877

